# Increased replication-associated single-stranded DNA promotes formaldehyde-induced mutagenesis in *Saccharomyces cerevisiae*

**DOI:** 10.1093/genetics/iyag189

**Published:** 2026-07-21

**Authors:** Thomas Blouin, Charlotte McGuinness, Kierra Marshall, Caroline Bazzle, Natalie Saini

**Affiliations:** Department of Biochemistry and Molecular Biology, Medical University of South Carolina, Charleston, SC 29425, United States; Department of Biochemistry and Molecular Biology, Medical University of South Carolina, Charleston, SC 29425, United States; Department of Biochemistry and Molecular Biology, Medical University of South Carolina, Charleston, SC 29425, United States; Department of Biochemistry and Molecular Biology, Medical University of South Carolina, Charleston, SC 29425, United States; Department of Biochemistry and Molecular Biology, Medical University of South Carolina, Charleston, SC 29425, United States

**Keywords:** formaldehyde, DNA repair, DNA replication, translesion synthesis, template switching, DNA–protein crosslink repair, mutagenesis, DNA damage

## Abstract

Formaldehyde (FA) is an environmentally abundant and endogenously produced aldehyde that has been shown to cause DNA damage, mutagenesis, and carcinogenesis. Several studies have demonstrated that FA induces guanine mutations resulting in a mutation signature like SBS40. In this work, we use *Saccharomyces cerevisiae* to demonstrate that replication defects generating single-stranded DNA (ssDNA) caused by the downregulation of the major replicative polymerases results in elevated FA mutagenesis. We found that loss of Mrc1 (CLASPIN) resulted in a high accumulation of ssDNA and FA mutagenesis, and that these phenotypes were not dependent on Mrc1's checkpoint activity. Loss of DNA–protein crosslink repair results in elevated FA sensitivity with no alteration to mutagenesis, likely due to the inability of the fork to bypass unprocessed protein adducts. Finally, we show that FA-induced mutagenesis is dependent on Pol ζ-mediated translesion synthesis, while deficiencies in the template switching pathway do not alter error-free bypass of FA adducts. Overall, our work points toward replication-associated ssDNA as a major substrate for FA-induced damage and elucidates the pathways that function to prevent FA mutagenesis at replication forks.

## Introduction

Formaldehyde (FA) is an environmentally abundant aldehyde and produced by cells endogenously through processes like 1-carbon metabolism and histone demethylation (Porter et al. 1985; Garrow et al. 1993; Aravind and Koonin 2001; Shi et al. 2004; Tsukada et al. 2006; Kikuchi et al. 2008; Morellato et al. 2021; Vijayraghavan and Saini 2023; Thapa and Chan 2025). Studies in rodents have shown that FA consumption either through inhalation or drinking water directly causes forestomach papilloma, leukemias, and gastrointestinal tract tumors (Kawanishi et al. 2014). Human population studies have also shown that occupational FA exposure is associated with increased mortality from myeloid leukemias and nasopharyngeal cancers (Coggon et al. 2003; Pinkerton et al. 2004; Beane Freeman et al. 2009; Hauptmann et al. 2004, 2009; Thapa and Chan 2025). For these reasons, the International Agency for Research on Cancer has deemed FA a class I carcinogen (Kawanishi et al. 2014).

Cellular tolerance to FA falls broadly into two tiers, detoxification of the aldehyde and repair of its adducts (Dingler et al. 2020). Essential to FA detoxification are the enzymes *ALDH2* and *ADH5*, and loss of these enzymes in mice has been shown to lead to disruptions in hematolymphoid development (Dingler et al. 2020). Aberrant ALDH activity or expression has been found in breast cancer, colorectal cancer, lung cancer, head and neck squamous cell carcinoma, prostate cancer, pancreatic cancer, bladder cancer, and glioblastoma (Xia et al. 2023). High expression in breast cancer, head and neck squamous cell carcinoma, cervical cancer, and ovarian cancer has been associated with poor overall survival (Xia et al. 2023). It is likely that these cancers, due to higher metabolic requirements and proliferation, are required to upregulate these enzymes to tolerate larger endogenous aldehyde loads.

FA is a highly reactive electrophile that can form a variety of adducts on DNA including direct base adducts (mostly N^2^-HOMeG and N^6^-HOMeA), DNA interstrand crosslinks, and DNA intrastrand crosslinks (Beland et al. 1984; Zhong and Que Hee 2005; Cheng et al. 2003, 2008; Moeller et al. 2011; Lu et al. 2012). FA is also known for its ability to form DNA–protein crosslinks (DPCs), most often with lysine and cysteine residues (Lu et al. 2010; Blouin and Saini 2024). Cells resolve this damage through a variety of DNA repair pathways, including base excision repair, nucleotide-excision repair, and homologous recombination (Kawanishi et al. 2014). Additionally, cells have evolved the ability to process DPCs directly at replication forks through the Wss1 (SPRTN) metalloprotease, further guarding against FA-induced genomic instability (Duxin et al. 2014; Stingele et al. 2014; Lopez-Mosqueda et al. 2016; Vaz et al. 2016; Yang et al. 2017; Reinking et al. 2020a; Ruggiano and Ramadan 2021; Perry and Ghosal 2022; Noireterre et al. 2023). Interestingly, coupling of detoxification defects with defects in the Fanconi Anemia pathway that processes interstrand crosslinks results in the development of acute T-cell leukemia (Langevin et al. 2011; Rosado et al. 2011; Garaycoechea et al. 2012; Pontel et al. 2015; Dingler et al. 2020).

Single-stranded DNA (ssDNA) is generated throughout the cell cycle and dysregulated accumulation of ssDNA poses a serious risk to genomic stability in the presence of a genotoxic agent. Because ssDNA lacks a template strand required for most canonical repair, DNA adducts formed within ssDNA are often bypassed by the DNA damage tolerance pathways translesion synthesis (TLS) and template switching (TS) whereby cells can directly replicate past damage (Prakash et al. 2005; Edmunds et al. 2008; Makridakis and Reichardt 2012; Sale 2013; Malik et al. 2020). Accordingly, various studies have shown that UV (Burch et al. 2011), enzymatic deamination (Chan et al. 2015), alkylating damage (Yang et al. 2010; Roberts et al. 2012; Sakofsky et al. 2014; Saini et al. 2020; Hudson et al. 2023), and oxidative damage (Degtyareva et al. 2013, 2019) can all target ssDNA and produce unique mutational patterns (Saini and Gordenin 2020). Given that ssDNA lacks template-directed repair and exposes the highly reactive primary amine of guanine and adenine to FA, ssDNA presents as a particularly susceptible genomic substrate for FA-induced mutagenesis (Kawanishi et al. 2014).

Prior work from our lab and others used an inducible subtelomeric ssDNA system in yeast to show that acute FA treatment caused an abundance of guanine mutations within ssDNA regions (Thapa et al. 2022; Blouin et al. 2025). FA-induced mutagenesis within ssDNA was shown to depend on TLS, and loss of TLS resulted in chromosomal arm loss events (Blouin et al. 2025). A major source of endogenous ssDNA in cells is DNA replication and arises when the CMG helicase unwinds the double helix at replication forks (Moyer et al. 2006; Bochman et al. 2008; Ilves et al. 2010; Coster et al. 2014; Noguchi et al. 2017; Douglas et al. 2018; Seo and Kang 2018; Sparks et al. 2019; Lewis et al. 2022). Typically, generation of ssDNA at the fork is tightly regulated by the intra-S-phase checkpoint whose main signaling kinase Mec1 (ATR) becomes activated by the accumulation of ssDNA-bound RPA and works to suppress origin firing and slow replication progression (Santocanale and Diffley 1998; Doksani et al. 2009; Zeman and Cimprich 2014; Iyer and Rhind 2017). Loss of this pathway or alterations to the normal replisome composition, like replicative polymerase downregulation or loss of critical replisome proteins (Mrc1/CLASPIN, Tof1/Timeless, Csm3/Tipin), have been shown to cause fork dysregulation resulting in abundant replication-associated ssDNA that is prone to breakage and/or mutagenesis (Osborn and Elledge 2003; Nedelcheva et al. 2005; Tourrière et al. 2005; Lou et al. 2008; Roberts et al. 2012; Bhagwat et al. 2016; Hoopes et al. 2016; Grabarczyk 2020; Sui et al. 2020).

While our prior work established FA as a ssDNA mutagen and described how TLS and DPC repair coordinate to bypass FA-induced DNA damage, it is currently unknown which sources of endogenously produced ssDNA may be targeted for FA-induced mutagenesis. In this study, we investigated ssDNA generated at dysregulated DNA replication forks as a target for FA-induced mutagenesis, as well as the pathways protecting replication forks from FA-induced genomic instability. Overall, we find that fork dysregulation significantly increases FA-induced mutagenesis. Mrc1's coupling function is required for preventing mutagenesis, while intra-S-phase checkpoint signaling is required for cellular survival following FA exposure. Finally, TLS but not TS contributes to the bypass of FA-induced adducts. Our work provides further insight as to which genomic substrates are at an elevated risk for aldehyde-induced genotoxicity.

## Materials and methods

### Yeast strains

The strains used in this study were derived from ySR128 with the genotype *MATa*, *ura3Δ*, *can1Δ*, *ade2Δ*, *ura3Δ*, *his7-2*, *leu2-3*, and *112 trp1-289.* The genes *CAN1*, *URA3*, and *ADE2* were reintroduced at the *LYS2* locus as the array *lys2::ADE2-URA3-CAN1*at a midchromosomal position of chromosome II (Roberts et al. 2012). Genetically modified yeast used in this study were generated using homology-directed insertion of dominant drug resistance cassettes as previously described (Goldstein and McCusker 1999). Strains were validated using PCR. The strains, primers, and plasmids used in this study can be found in Supplementary Table 1. Strains carrying the *MRC1-AQ* allele are the same as described previously (Osborn and Elledge 2003).

### RT-qPCR of polymerase downregulation strains

Cultures of wildtype and *TetR-POL1*yeast were grown in yeast extract, peptone, and dextrose (YPD) or YPD containing 4 µg/mL doxycycline (TCI America) overnight. The following day, these cultures were subcultured in fresh YPD and doxycycline for ∼4 h and RNA was extracted using the Monarch Spin RNA Isolation Kit (New England BioLabs). RNA was quantified on a NanoDrop 1000 (Thermo Scientific) and quality assessed on a 1% agarose gel. Polymerase downregulation was measured via RT-qPCR using the New England Biolabs Luna Universal One-Step RT-qPCR kit. *ACT1* expression was used for normalization, generating ΔCt values. Polymerase downregulation strains grown in YPD were compared to the wildtype strain grown in YPD, and polymerase downregulation strains grown in YPD with doxycycline were compared to wildtype in doxycycline, producing ΔΔCt values. Fold-change in gene expression was then calculated as 2^*−*ΔΔCt^. Gene expression levels of wildtype cultures were denoted as a fold-change of 1, and polymerase downregulation strains were compared against them. Primers used for RT-qPCR are described in Supplementary Table 1b. RT-qPCR data for *TetR-POL2*and *TetR-POL3*were from Porcher et al. (2025), and primers used for these reactions can be found therein (Porcher et al. 2025).

### Fluctuation assay for measuring FA-induced mutagenesis

Strains carrying the *lys2::ADE2-URA3-CAN1*reporter array were streaked-out onto YPD (+4 µg/mL doxycycline for *POL* downregulation) plates or YPD plates containing 2 mM FA (Electron Microscopy Sciences) (+4 µg/mL doxycycline for Pol downregulation) and were incubated at 30˚C for 72 h. Individual colonies from the streak outs were patched onto fresh YPD plates with and without 2 mM FA and incubated for 24 h. Two independent wildtype isolates were used to generate 6 patches, while two independent experimental isolates were used to generate 12 patches. Patches were resuspended in 1 mL of sterile water and used to create a dilution series. The 5th and 6th dilutions were plated onto synthetic complete (SC) media to determine the number of cells present in each patch, and the 0th dilution was plated onto SC-Arginine plates containing 60 mg/mL canavanine (Sigma) and 20 mg/mL adenine to isolate Can^R^ colonies. Plates were incubated at 30˚C for 72 h and colonies were counted using an aCOLyte3 (Synbiosis Inc.).

Mutation rates were calculated as previously described (Saini et al. 2017). In brief, the mutation rate (µ) was calculated based on the mutant frequency (*f*) and the total number of cells in a culture (*N*) resolving by iterations the previously described transcendent equation μ=0.43f/log(N×μ) (Drake 1991). These calculations were performed in excel using an established macro which can be made available upon request. The highest and lowest mutation rates within an experimental group were removed to account for jackpot events and the remaining rates were used to calculate the median. Mutation rate figures show the mutation rate of each patch as a data point. The median is depicted as a horizontal bar and error bars depict 95% confidence interval. *P*-values were calculated using a Mann–Whitney unpaired *U*-test and multiple testing correction was performed by analyzing stacked *P*-values.

### Spot dilutions

Yeast strains were resuspended in water to a concentration of 1 × 10^7^ cells/mL. 200 µL of this solution was placed into the top wells of a 96-well plate. This solution was then diluted 10-fold per row of the spot dilution. 3 µL of solution from each well was spotted onto YPD, YPD with 4 µg/mL doxycycline, YPD with 1 or 2 mM FA, or YPD with both doxycycline and FA. Spots were grown at 30˚C for 48 h and imaged on aCOLyte3.

### Clamped homogenous electric field electrophoresis

Clamped homogenous electric field electrophoresis (CHEF) gels were produced as previously described (Wei 2006) in chapter 8 (PMID: 16118425). Strains were grown overnight in YPD or YPD containing 4 µg/mL doxycycline. The following day, cells were subcultured into a fresh 50 mL of YPD with or without doxycycline and with or without 2 mM FA for ∼5 h. Approximately 5 × 10^7^ cells were taken from each culture to create the agarose plugs. S1 digestion was performed the day the CHEF was ran by cutting one plug in half. Both halves were washed with S1 buffer (0.2 M NaCl, 50 mM sodium acetate, 1 mM M zinc sulfate, 0.5% glycerol, pH 4.5) for 30 min 3 times. To one of the plugs, 500 U/mL S1 nuclease (Promega) was added, and both plugs were incubated at 37˚C for 1.5 h. For pulsed-field gel electrophoresis, a 1% agarose gel in 0.5 × TBE was used and ran on a BioRad CHEF Mapper at a temperature of 14˚C with switch time 60–120 s, run time 24 h, angle 120˚, and voltage gradient of 6 V/cm. Gels were stained with ethidium bromide and visualized on a BioRad GelDoc Go imaging system. ImageJ (Version 1.54p) was used to generate the CHEF intensity plots. This was done by inverting the background of the CHEF gel and using the rectangle tool to highlight lane 1 of the gel. The dimensions of this box were used to analyze all subsequent lanes. Once a lane was defined, the plot lanes feature was used and then analyzed line graphs to get a .csv file of intensity values along the lane. The final intensity plots were generated in R.

## Results

### Downregulation of replicative polymerases increases FA-induced mutagenesis and sensitivity

*Saccharomyces cerevisiae*, like mammalian cells, utilize Pol ε, Pol δ, and Pol α to faithfully replicate the genome (Kawasaki and Sugino 2001). To begin probing the effect of FA on replication-associated ssDNA, we first generated *S. cerevisiae* with dysfunctional replication by replacing the endogenous promoters of *POL2* (catalytic subunit of the leading strand Pol ε), *POL3* (catalytic subunit of primarily lagging strand Pol δ), and *POL1* (catalytic subunit of Pol α) with tetracycline repressible *tetO_7_* promoters (Bellí et al. 1998). RT-qPCR of *POL2* and *POL1* showed expression was reduced to ∼50%, and *POL3* expression was found to be reduced to ∼30% 4 h after doxycycline addition (Supplementary Fig. 1, Supplementary Table 2) (Porcher et al. 2025). Use of the same *tetO_7_* promoter has been previously shown to result in a 10-fold reduction in protein levels of Pol3 (Saini et al. 2013).

Spot dilutions were used to assess the sensitivity of polymerase downregulation strains to FA. We did not observe any reduction in viability of yeast strains with replicative polymerase downregulation, as is evident on the YPD with doxycycline plate (Fig. 1a). At 2 mM FA, however, *TetR-POL2*strains appear slightly sensitive compared to the wildtype (Fig. 1a). *TetR-POL3*and *TetR-POL1*display no apparent sensitivity to FA (Fig. 1a).

**Fig. 1.**
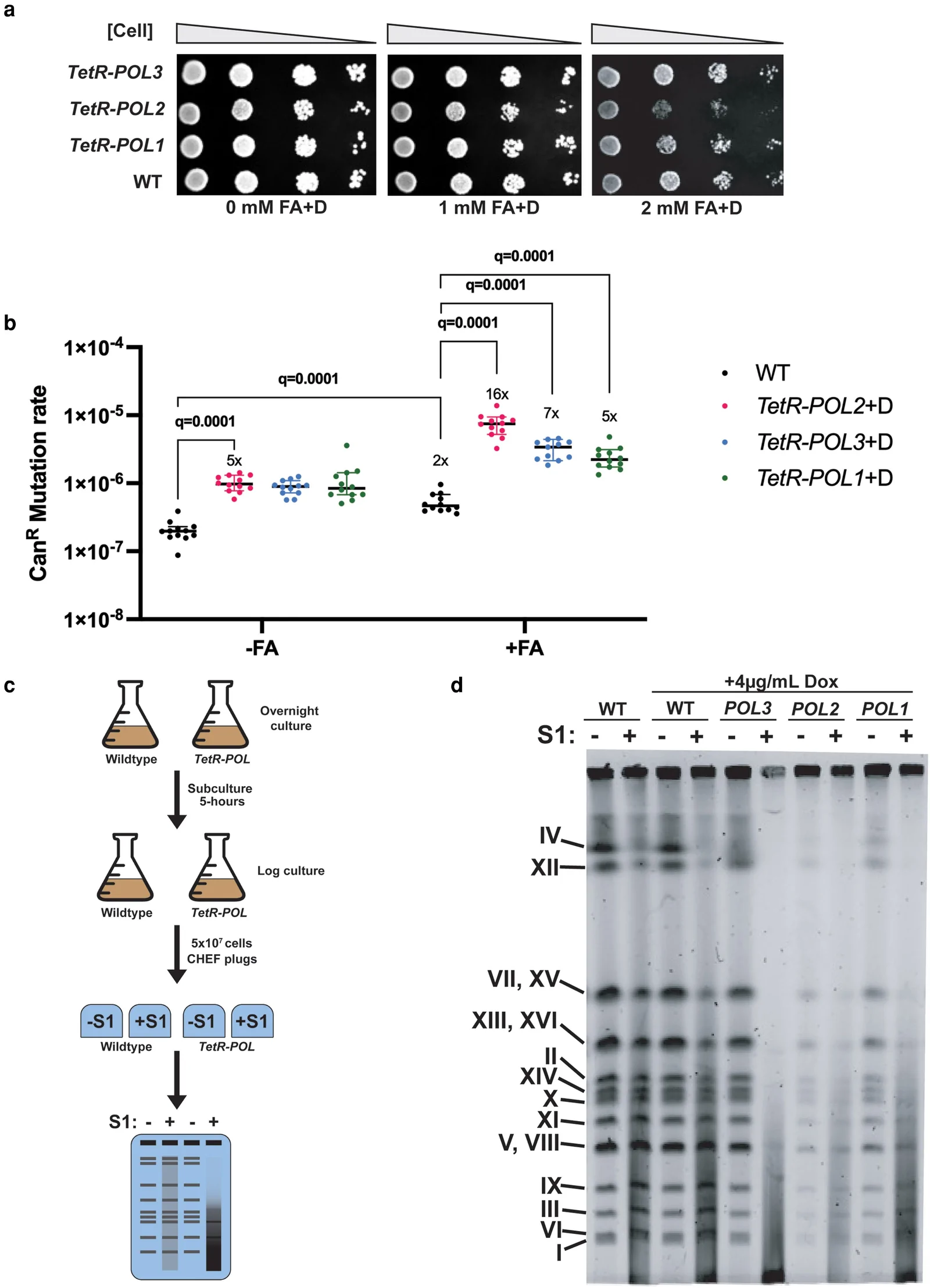
Downregulation of replicative polymerases increase formaldehyde-induced mutagenesis and sensitivity. a) Spot dilutions of replicative polymerase downregulation strains grown in the presence of 4 µg/mL doxycycline and various concentrations of FA for 2 d. b) Can^R^ mutation rate of various replicative downregulation strains when chronically exposed to 2 mM FA (+FA) or YPD (−FA) and 4 µg/mL doxycycline for 4 d. Bars represent median with 95% CI, and *Y*-axis is plotted on a log scale. *P*-values were calculated using a Mann–Whitney unpaired *U*-test and multiple testing correction was performed by analyzing stacked *P*-values. All significant changes with a greater than 2-fold difference are depicted on the graph. c) Experimental design for ssDNA detection using S1 nuclease and CHEF. All *TetR-POL* strains were grown in the presence of 4 µg/mL doxycycline. The representative CHEF gel shows expected results for a strain with low ssDNA in lane 2 and a strain with high ssDNA in lane 4. d) Representative CHEF gel of wildtype or *POL* downregulation strains. ± corresponds to addition of the ssDNA specific S1 nuclease.

We further assayed FA-induced mutagenesis in each of these strains. The wildtype yeast strains demonstrated a 2-fold increase in the Can^R^ mutation rate upon FA exposure (Fig. 1b, Supplementary Table 3). This suggests that in the absence of abnormal levels of ssDNA, FA is weakly mutagenic to *S. cerevisiae*. This assay was repeated using the polymerase downregulation strains. In the absence of FA, we observed significant 5-, 5-, and 4-fold increases in the *TetR-POL2*, *TetR-POL3*, and *TetR-POL1*strains, respectively, over wildtype (Fig. 1b, Supplementary Table 3). These data support several reports that depletion of major replicative polymerases and overall dysregulation of replication forks results in elevated genome instability (Lemoine et al. 2005; Zheng et al. 2016; Mertz et al. 2017). Upon treatment with 2 mM FA and doxycycline, *TetR-POL2*strains showed a 16-fold increase in the Can^R^ mutation rate over FA-treated treated wildtype strains and an 8-fold increase over the untreated *TetR-POL2*strains (Fig. 1b, Supplementary Table 3). Similarly, *TetR-POL3*strains exposed to FA and doxycycline showed a 7-fold increase in mutation rate over FA-treated wildtype strains and a 4-fold increase over untreated *TetR-POL3*strains (Fig. 1b, Supplementary Table 3). Finally, FA and doxycycline-treated *TetR-POL1*strains showed a 5-fold increase above FA-treated wildtype isolates and a 3-fold increase above untreated *TetR-POL1*strains (Fig. 1b, Supplementary Table 3). Taken together, these results suggest that FA is highly mutagenic in strains with dysfunctional replication forks.

To confirm ssDNA was being generated in the polymerase downregulation strains, we analyzed genomic DNA via contour CHEF, wherein intact chromosomes can be separated as distinct bands. We used log-phase cultures growing in the presence of doxycycline to make CHEF plugs (Fig. 1c). Each plug was split and half was incubated with S1 nuclease which has been shown to have 10,000 × higher nuclease activity on ssDNA than dsDNA for 1.5 h (Desai and Shankar 2003). Cleavage of chromosomes at replication-associated ssDNA gaps is expected to appear as a smear in the gels. S1 treatment of wildtype strains grown in either YPD or YPD supplemented with doxycycline resulted in minimal smearing on the gel and overall conservation of band peaks in the intensity plots, showing that wildtype strains generate low levels of ssDNA (Fig. 1d, Supplementary Fig. 2a–d). In contrast, each of the polymerase downregulation strains showed extensive smearing and loss of chromosomal banding pattern upon treatment with S1 nuclease (Fig. 1d, Supplementary Fig. 2e–j). Interestingly, CHEF analysis of 2 mM FA-treated isolates did not demonstrate a detectible increase in ssDNA above what was observed in the untreated wildtype or *TetR-POL2*strains (Supplementary Fig. 3a and b). These observations indicate that FA is likely not the source of ssDNA in the replication defective strains but rather causes damage to pre-existing ssDNA.

While the *TetR-POL2*and *TetR-POL1*strains grown in YPD containing doxycycline maintained a banding pattern like wildtype, we noted that the chromosome bands were consistently fainter than wildtype (Fig. 1d, Supplementary Fig. 3a). It is possible that while these strains do not have long-term viability defects (Fig. 1a), they may experience slower growth than the *TetR-POL3*or wildtype strains, potentially due to increased fork stalling and ssDNA accumulation. We did not see this decrease in chromosome intensity in the *TetR-POL3*strains, potentially indicating lower levels of ssDNA, replication stalling, and branched intermediates being formed in these strains as compared to *TetR-POL2*and *TetR-POL1*strains. Overall, these results show that downregulation of the replicative polymerases results in higher ssDNA levels than what is observed in wildtype strains.

### Mrc1 function in replisome coupling is required to prevent FA-induced mutagenesis

Mrc1 is an integral component of the replication fork protection complex with dual roles as both a signaling adapter protein in the intra-S-phase checkpoint and as a physical link between Pol ε and the CMG helicase (Alcasabas et al. 2001; Katou et al. 2003; Osborn and Elledge 2003; Nedelcheva et al. 2005; Szyjka et al. 2005; Tourrière et al. 2005; Gambus et al. 2006; Hodgson et al. 2007; Lou et al. 2008; Chen and Zhou 2009; Pardo et al. 2017; Yeeles et al. 2017). To investigate the role of Mrc1 in FA-induced mutagenesis, we performed fluctuation assays in the presence or absence of 2 mM FA. Similarly to the polymerase downregulation strains, we observed a 5-fold increase in mutagenesis in the untreated *mrc1Δ* strains when compared to the untreated wildtype, suggesting that loss of Mrc1 results in dysregulated forks (Fig. 2a, Supplementary Table 3). After chronic exposure to 2 mM FA, *mrc1Δ* strains were found to have a significant 10-fold increase in the Can^R^ mutation rate when compared to FA-treated wildtype strains, and a 5-fold increase in mutation rate over the untreated *mrc1Δ* strain (Fig. 2a, Supplementary Table 3). To determine whether Mrc1's coupling or signaling function was required to prevent FA-induced mutagenesis, we reintroduced a previously described *MRC1-AQ* mutant allele into the *mrc1Δ* background (Osborn and Elledge 2003). Importantly, this mutant can localize to the replication fork and maintain coupling between Pol ε and the CMG helicase, but is unable to be activated by Mec1 (ATR) upon replication stress (Osborn and Elledge 2003). We found that mutation rates were not elevated in either the untreated Mrc1-AQ strains or following chronic exposure to 2 mM FA (Fig. 2a, Supplementary Table 3). Rad9 (53BP1) is another DNA damage checkpoint adapter protein that is typically activated by Tel1 (ATM) in response to double-stranded breaks but can compensate for loss of Mrc1 signaling leading to downstream Rad53 activation (Siede et al. 1993; Emili 1998; Gilbert et al. 2001; Sweeney et al. 2005; Pfander and Diffley 2011; Bacal et al. 2018). To ensure that the reduction in mutation rate seen in *MRC1-AQ* strains was not because of Rad9 compensation, we generated *rad9Δ* and *MRC1-AQ–rad9Δ* strains. We observed Can^R^ mutation rates like wildtype in *rad9Δ* isolates, and only a modest 2-fold increase in *MRC1-AQ–rad9Δ* isolates exposed to FA (Fig. 2a, Supplementary Table 3). These results suggest that the ability of Mrc1 to maintain a coupled replisome is responsible for protecting against FA-induced mutagenesis.

**Fig. 2.**
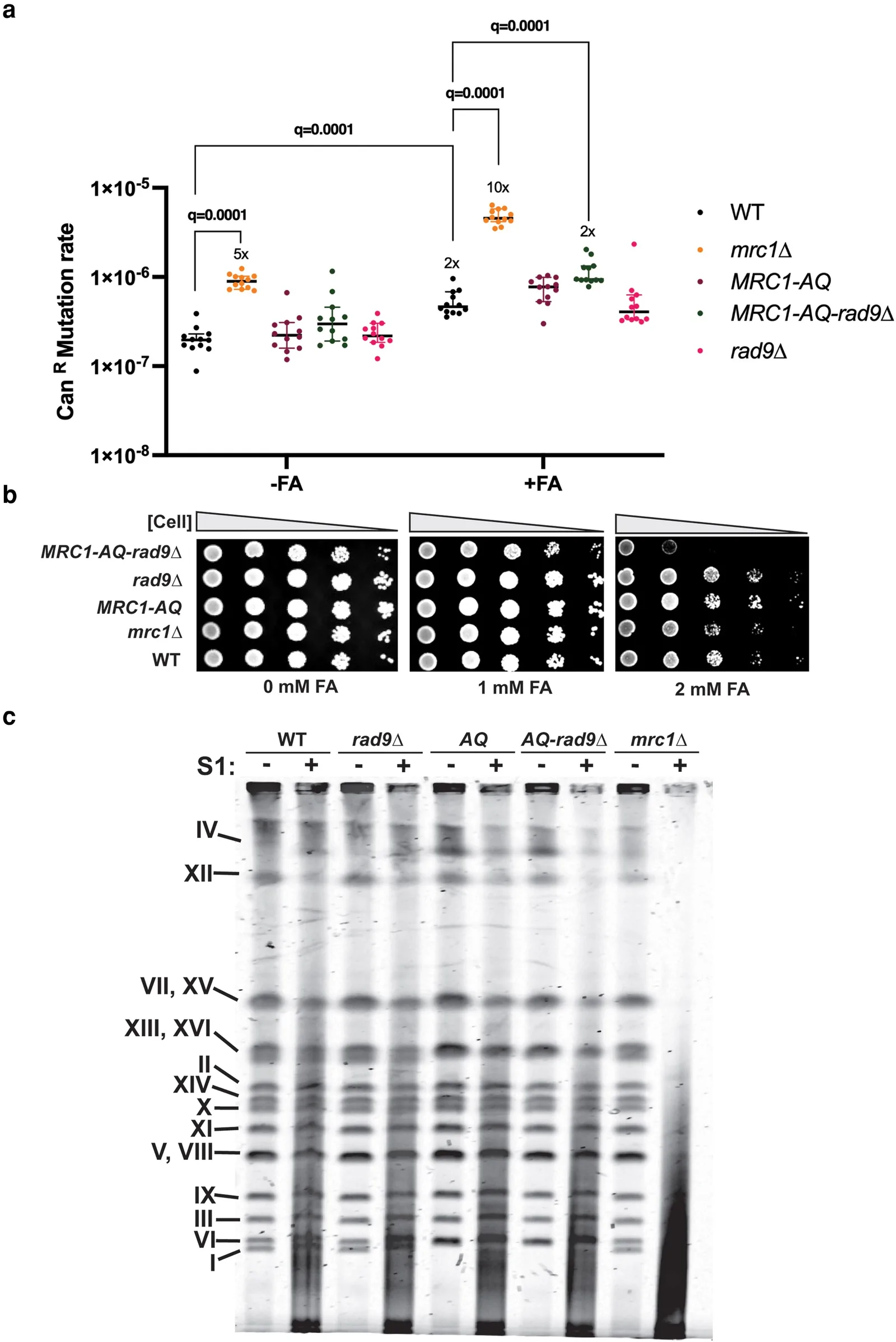
Mrc1 function in replisome coupling is required to prevent formaldehyde-induced mutagenesis. a) Can^R^ mutation rate of DNA damage checkpoint adapter protein-deficient strains when chronically exposed to 2 mM FA (+FA) or YPD (−FA) for 4 d. Bars represent median with 95% CI, and the *Y*-axis is plotted on a log scale. *P*-values were calculated using a Mann–Whitney unpaired *U*-test. Multiple testing correction was performed by analyzing stacked *P*-values. All significant changes with a greater than 2-fold difference are depicted on the graph. b) Spot dilutions of checkpoint mutant strains grown in the presence of various concentrations of FA for 2 d. c) Representative CHEF gel of wildtype or checkpoint mutant strains. ± corresponds to addition of the ssDNA specific S1 nuclease. *AQ* corresponds to *MRC1-AQ* isolates.

Next, we asked if strains with ablated Mrc1 or Rad9 function were sensitive to FA. At 2 mM FA, *mrc1Δ* strains were observed to have a modest sensitivity relative to the wildtype (Fig. 2b). *MRC1-AQ* and *rad9Δ* strains displayed no sensitivity, while the *MRC1-AQ*–*rad9Δ* strain displayed significantly heightened sensitivity (Fig. 2b). This result supports the idea that Mrc1 and Rad9 signaling works in parallel to maintain survival during FA exposure, likely by allowing efficient repair of FA-induced DNA adducts. Overall, our results suggest that Mrc1's role in maintaining replisome coupling is essential in preventing FA-induced mutagenesis, and that DNA damage checkpoint signaling either via Mrc1 or Rad9 is required for cells to tolerate FA.

Finally, we assessed ssDNA levels in Mrc1 or Rad9 defective strains. We observed wildtype-like banding patterns in *rad9Δ*, *MRC1-AQ*, and *MRC1-AQ–rad9Δ* strains treated with S1, demonstrating that loss of checkpoint signaling adapters does not lead to accumulation of ssDNA (Fig. 2c and Supplementary Fig. 4a–h). In contrast, we observed significant loss of the banding pattern and smoothing of the peak intensity profile in *mrc1Δ* strains, as expected given the role of Mrc1 in replisome coupling (Fig. 2c and Supplementary Fig. 4i and j).

### DPC repair promotes cellular survival to FA exposure but has minor impacts on mutagenesis

FA has been shown to be a potent DPC (Merk and Speit 1998; de Graaf et al. 2009; Hoffman et al. 2015; Lai et al. 2016; Nakamura and Nakamura 2020; Blouin and Saini 2024). In yeast, Wss1 (SPRTN) is a critical component of the cellular response to DPCs and has been shown to associate at DNA replication forks (de Graaf et al. 2009; Duxin et al. 2014; Stingele et al. 2014; Lopez-Mosqueda et al. 2016; Vaz et al. 2016; Mórocz et al. 2017; Larsen et al. 2019; Reinking et al. 2020a, 2020b; Noireterre et al. 2023). To determine the impact of DPCs on FA-induced mutagenesis at dysregulated replication forks, we tested FA-induced mutagenesis in *wss1Δ*, *TetR-POL2-wss1Δ*, and *mrc1Δ-wss1Δ* strains using fluctuation assays. *wss1Δ* isolates showed no significant increase in mutagenesis above wildtype in untreated cultures or following chronic exposure to 2 mM FA (Fig. 3a, Supplementary Table 3). Spot dilutions revealed *wss1Δ* isolates to be significantly more sensitive to FA than wildtype isolates, underscoring the importance for Wss1 in cellular survival following FA exposure (Fig. 3b). *TetR-POL2-wss1Δ* isolates displayed a minor 2-fold increase in Can^R^ mutation rate above *TetR-POL2*in the absence of FA, but no further increase was observed in the *TetR-POL2-wss1Δ* when grown in the presence of FA (Fig. 3a, Supplementary Table 3). Similarly, *mrc1Δ-wss1Δ* isolates had a comparable mutation rate to *mrc1Δ* both in the absence and in the presence of FA (Fig. 3a, Supplementary Table 3). Both *TetR-POL2-wss1Δ* and *mrc1Δ-wss1Δ* isolates appeared to be more sensitive to FA than the single mutants (Fig. 3b). We conclude that an impaired ability to repair DPCs through loss of Wss1 has a minor impact on FA-induced mutagenesis both at wildtype and dysregulated replication forks. However, Wss1 is critical to cellular survival following FA exposure, resulting in a slight increase in sensitivity when DPC repair is lost in the context of dysregulated DNA synthesis.

**Fig. 3.**
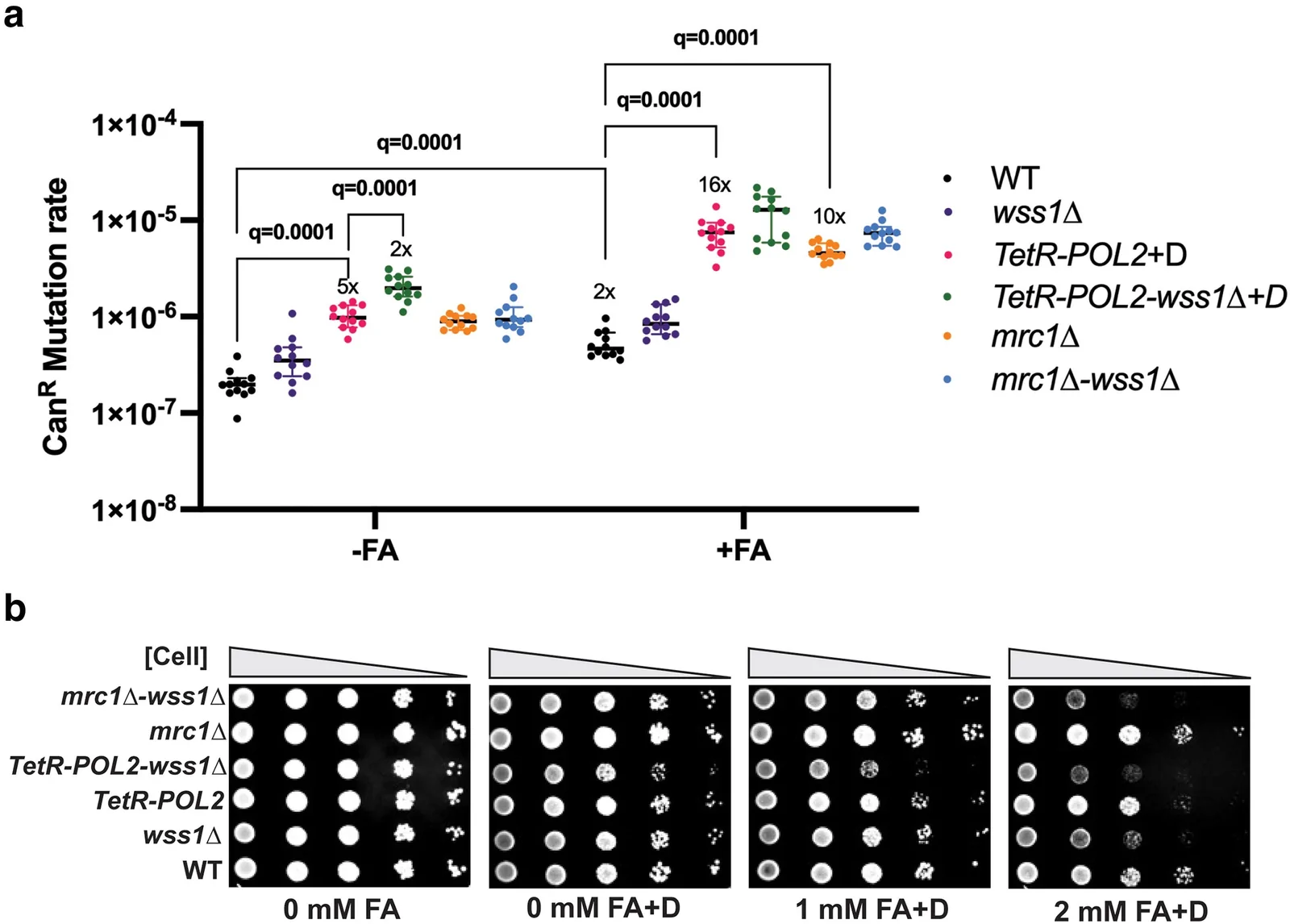
DNA–protein crosslink repair promotes survival following formaldehyde exposure but has minor impacts on mutagenesis. a) Can^R^ mutation rate of leading strand dysregulation and DPC repair defective strains when chronically exposed to 2 mM FA (+FA) or YPD (−FA) and 4 µg/mL doxycycline (*TetR-POL2* and *TetR-POL2-wss1Δ*) for 4 d. Bars represent median with 95% CI, and the *Y*-axis is plotted on a log scale. *P*-values were calculated using a Mann–Whitney unpaired *U*-test and multiple testing correction was performed by analyzing stacked *P*-values. All significant changes with a greater than 2-fold difference are depicted on the graph. b) Spot dilutions of leading strand dysregulation and DPC repair defective strains grown in the presence of 4 µg/mL doxycycline and various concentrations of FA for 2 d.

### TS is not involved in the error-free bypass of FA adducts

Because ssDNA is not compatible with templated repair pathways, cells are increasingly dependent on DNA damage tolerance pathways when adducts evade repair and stall the replisome (Frouin et al. 2003; Hedglin and Benkovic 2017; Bainbridge et al. 2021; Wong et al. 2021). TS uses homology on the complementary strand of an adjacent sister chromatid as the template for error-free DNA synthesis past an adduct (Ler and Carty 2021). Alternatively, TS may involve the reversal of a stalled replication fork where nascent strands anneal with one another (Chang and Cimprich 2009). TS depends on polyubiquitylation of PCNA, which is carried out by Rad5 (HLTF), Mms2, and Ubc13 (UBE2), and deletion of any of these factors has been shown to equally impair polyubiquitylation (Ulrich and Jentsch 2000; Hoege et al. 2002; Unk et al. 2006; Xu et al. 2016; Meister et al. 2025). To investigate the role of TS in bypassing FA-induced adducts, we generated *TetR-POL2-ubc13Δ*, *TetR-POL3-ubc13Δ*, and *ubc13Δ* strains. Loss of TS resulted in a significant 7-fold increase in the Can^R^ mutation rate in the absence of FA (Fig. 4a, Supplementary Table 3). Loss of TS in either fork dysregulation strain resulted in a further increase in the mutation rate of untreated cultures when compared to the fork dysregulation strain alone (Fig. 4a, Supplementary Table 3). Interestingly, deletion of *UBC13* both on its own and in the *TetR-POL2*and *TetR-POL3*backgrounds resulted in no significant increase in the Can^R^ mutation rate following FA exposure compared to untreated cultures (Fig. 4a, Supplementary Table 3). Spot dilutions revealed that loss of TS switching also did not increase sensitivity to FA (Fig. 4b). Taken together, these results suggest that while TS is important in the error-free bypass of some types of DNA damage, TS does not contribute to the bypass of FA-induced DNA adducts.

**Fig. 4.**
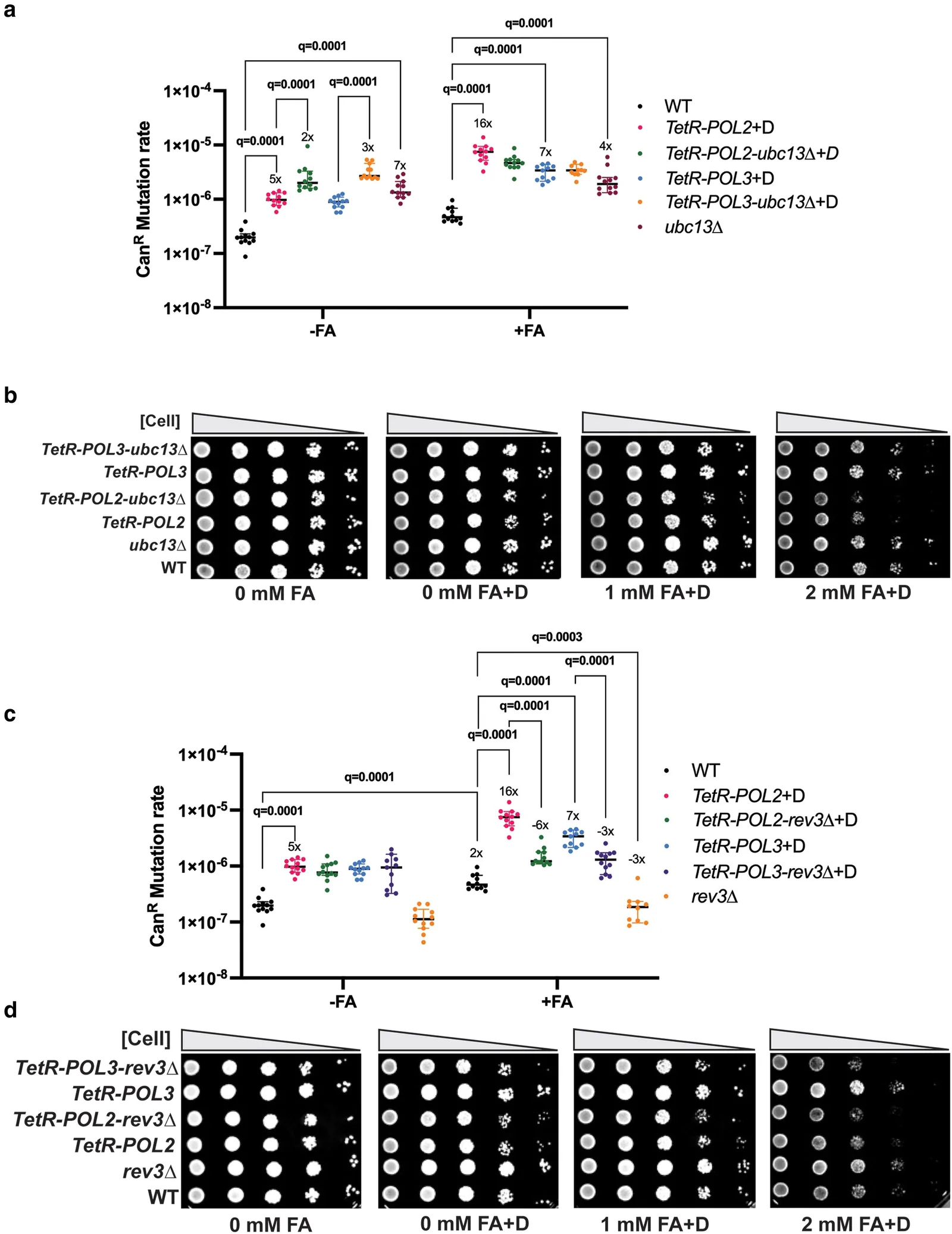
DNA damage tolerance pathways play differing roles in bypass of formaldehyde adducts. a) Can^R^ mutation rate of template switching deficient strains chronically exposed to 2 mM FA (+FA) or YPD (−FA) and 4 µg/mL doxycycline (*TetR-POL2*, *TetR-POL3*, *TetR-POL2-ubc13Δ*, and *TetR-POL3-ubc13Δ*) for 4 d. b) Spot dilutions of replication and template switching deficient strains grown in the presence of 4 µg/mL doxycycline and various concentrations of FA for 2 d. c) Can^R^ mutation rate of translesion synthesis deficient strains chronically exposed to 2 mM FA (+FA) or YPD (−FA) and 4 µg/mL doxycycline (*TetR-POL2*, *TetR-POL3*, *TetR-POL2-rev3Δ*, and *TetR-POL3-rev3Δ*) for 4 d. d) Spot dilutions of replication and translesion synthesis deficient strains grown in the presence of 4 µg/mL doxycycline and various concentrations of FA for 2 d. Bars in (a) and (c) represent median with 95% CI, *P*-values were calculated using a Mann–Whitney unpaired *U*-test and multiple testing correction was performed by analyzing stacked *P*-values. All significant changes with a greater than 2-fold difference are depicted on the graph.

### TLS is required for the error-prone bypass of FA-induced DNA adducts

To investigate the role of TLS in bypassing FA-induced adducts, we generated strains lacking Rev3, which is the catalytic subunit of Pol ζ (*rev3Δ*, *TetR-POL2-rev3Δ*, and *TetR-POL3-rev3Δ*). Loss of TLS bypass did not alter the mutation rate of untreated strains. In contrast, loss of Rev3 led to an overall reduction in mutagenesis upon FA treatment; *TetR-POL2-rev3Δ* strains had a 6-fold reduction in the Can^R^ mutation rate when compared to *TetR-POL2*, while *TetR-POL3-rev3Δ* strains displayed a 3-fold reduction in the Can^R^ mutation rate when compared to *TetR-POL3*(Fig. 4c, Supplementary Table 3). Finally, we observed that FA-treated *rev3Δ* strains had a 3-fold reduction in the mutation rate when compared to wildtype, suggesting that TLS is responsible for most FA-induced mutations even in the absence of ssDNA accumulation (Fig. 4c, Supplementary Table 3). These results agree with prior work from our lab showing that Pol ζ is required for the mutagenic bypass of FA-induced adducts within ssDNA and that in the absence of Pol ζ, persistent stalling due to inefficient TLS results in chromosome breakage events (Blouin et al. 2025)*. rev3Δ* strains did not appear any more sensitive to FA than wildtype (Fig. 4d). Interestingly, loss of TLS in the context of Pol2 or Pol3 depletion resulted in further sensitization to FA than what was observed in strains with intact TLS (Fig. 4d). This result suggests that while cells are able to tolerate loss of TLS in the absence of abundant ssDNA, loss of TLS during fork dysregulation increases FA sensitivity likely due to increased break events as was shown previously (Blouin et al. 2025). Overall, our results suggest that FA-induced mutagenesis at dysregulated DNA replication forks depends on error-prone TLS bypass mediated by Pol ζ.

## Discussion

FA has been shown in various studies to induce mutations and genome instability. In *S. cerevisiae*, a *lys2* frameshift reversion assay was used to show FA induces large, nucleotide-excision repair-dependent deletions and established a role for TLS in the bypass of FA-induced adducts (Grogan and Jinks-Robertson 2012; Vijayraghavan and Saini 2023). FA has been shown to mutagenize areas of ssDNA in yeast, resulting in an SBS40-like mutation signature that has also been observed in mice (Dingler et al. 2020; Thapa et al. 2022). Recently, our lab confirmed these findings and established that FA-induced adducts were forming predominantly on guanine residues in ssDNA (Blouin et al. 2025). Pol ζ-mediated TLS was required for mutational accumulation within ssDNA and in the absence of TLS; there was a significant increase in the frequency of chromosomal arm loss events (Blouin et al. 2025). Efficient repair of these arm loss events was dependent on the DPC protease Wss1, suggesting that DPCs are a major adduct type within areas of ssDNA (Blouin et al. 2025). However, the above study utilized a *cdc13-1* mutant to generate ssDNA at subtelomeric regions in yeast, resulting in a G2 arrest that allowed for an acute FA treatment. Further work is therefore needed to understand how these pathways may be involved in the FA-induced mutagenesis of endogenously generated ssDNA.

In this work, we demonstrate that replication-associated ssDNA is also highly susceptible to FA-induced mutagenesis. We find that dysregulation of DNA replication through the downregulation of the *POL1*, *POL2*, and *POL3* genes or deletion of *MRC1* causes an increase in ssDNA accumulation. Moreover, downregulated *POL1*, *POL2*, *POL3*, and *mrc1Δ* strains experienced elevated FA-induced mutagenesis. Such mutagenicity of ssDNA is likely due to the exposure and damage of primary amines typically protected within the interior of the helix, resulting in FA-induced lesion formation. These adducts typically persist due to the lack of template-directed repair and must thus be bypassed to prevent prolonged polymerase stalling (Fig. 5). Interestingly, we observed a stronger genotoxicity of FA in strains with *POL2* downregulation than with downregulated *POL3* or *POL1* (Fig. 1a and b). This increased mutagenic effect on the leading strand is potentially explained by the different repriming efficiencies of the leading and lagging strands.

**Fig. 5.**
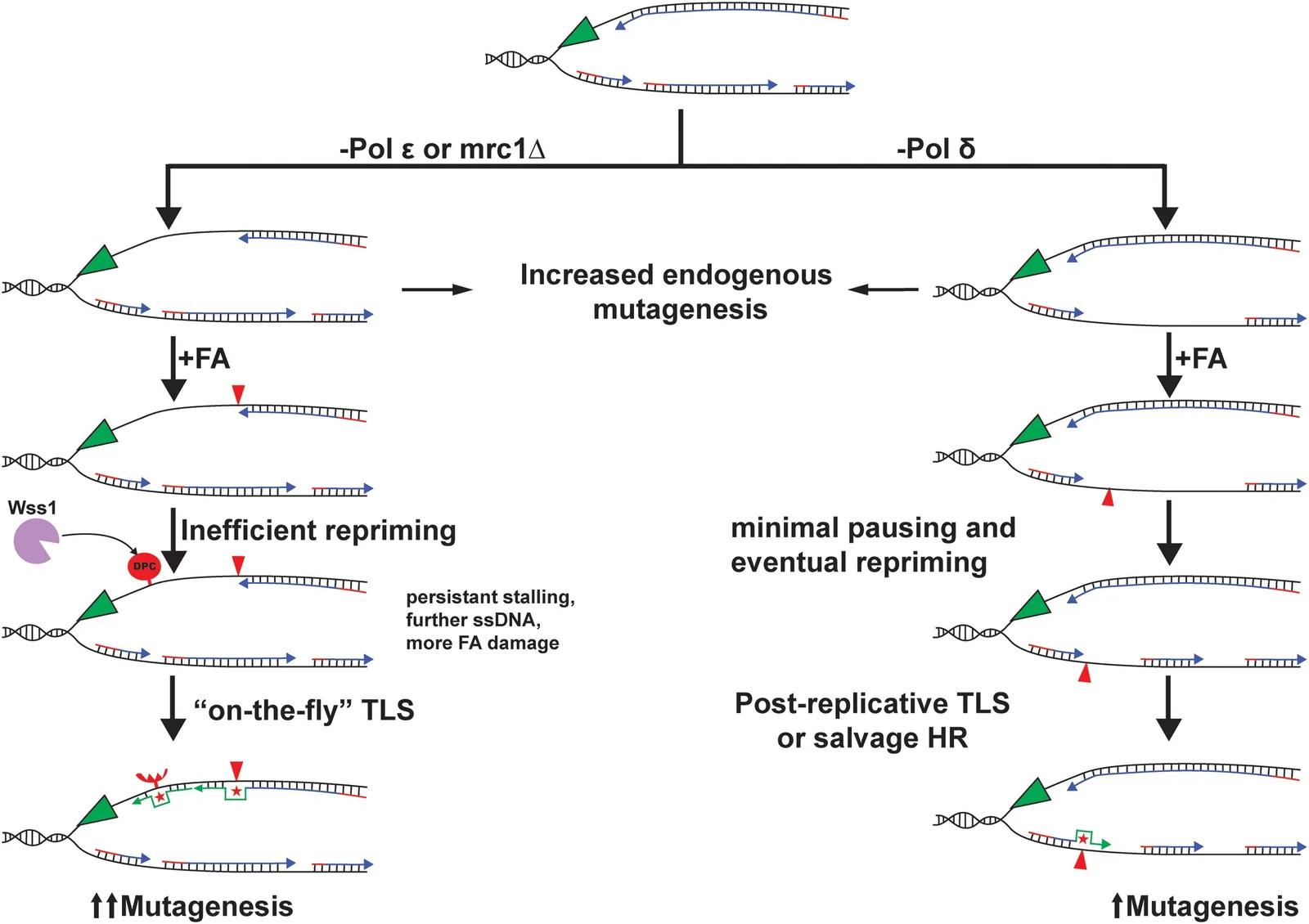
Proposed model for formaldehyde-induced mutagenesis at dysregulated leading and lagging strands. Downregulation of the replicative polymerases increases the amount of replication-associated ssDNA, and this dysregulation increases endogenous mutations. Leading strand adducts stall replication until TLS or repriming can occur. Because repriming of the leading strand is thought to be inefficient, leading strands exhibit a greater dependency on “on-the-fly” TLS or risk further uncoupling, more damage accumulation, and higher mutation rates. Adducts on the lagging strand also stall replication, but efficient repriming limits the generation of ssDNA at the fork, thus favoring a postreplicative model of TLS.

Eukaryotic leading strands are typically synthesized continuously from one RNA–DNA primer hybrid, limiting the need for repriming (Burgers and Kunkel 2017). Upon collision with a Pol ε stalling lesion, the damage must either be bypassed by a DNA damage tolerance pathway or the leading strand may be reprimed downstream of the lesion leaving a daughter-strand gap to be dealt with in postreplicative repair (Bainbridge et al. 2021; Wong et al. 2021). While higher eukaryotes have acquired the primase PrimPol to more efficiently restart stalled leading strands, yeast lack this protein (García-Gómez et al. 2013). As such, reconstitution of the yeast replisome in vitro has revealed that leading strand repriming past an adduct is exceedingly rare and inefficient, likely placing a larger responsibility on “on-the-fly” DNA damage tolerance pathways to ensure stalling is temporary (Fig. 5) (Taylor and Yeeles 2018, 2019). Inefficient or delayed repriming of the leading strand has been shown to leave significantly larger ∼1,600 nucleotide long gaps when compared to ∼200 nucleotide long gaps on the lagging strand (Lopes et al. 2006; Hedglin and Benkovic 2017). Thus, potentially more FA-induced adducts could form on the leading strand resulting in the higher FA-induced mutation rate observed in *POL2* downregulation strains (Figs. 1b and 5). Further, lack of TLS would result in fork collapse and cell death (Fig. 4d). Lagging strands, however, are synthesized discontinuously from many RNA–DNA primers, generating Okazaki fragments that must be ligated (Burgers and Kunkel 2017). As such, repriming on the lagging strand happens quickly and limits the amount of uncoupling experienced (Taylor and Yeeles 2018). This would result in smaller stretches of ssDNA and thus lower FA-induced toxicity and mutagenicity (Fig. 1a and b). Depletion of Pol α has been shown to result in significantly longer Okazaki fragments on the lagging strand and an overall global decrease in replication origin firing efficiency (Porcella et al. 2020). This overall reduction in origin firing could limit the generation of ssDNA and potentially explains why Pol α strains had the lowest Can^R^ mutation rate in our assay (Fig. 1b).

TS and TLS are critical DNA damage tolerance pathways that limit or overcome fork stalling (Bainbridge et al. 2021; Wong et al. 2021). In this study, we find that TS plays a minor role in bypassing FA adducts, while TLS plays a critical role in the error-prone bypass of FA-induced DNA damage (Fig. 4). TS has been shown to bypass a variety of adducts, including UV damage, alkylation damage, and intrastrand crosslinks in an error-free manner (Fujiwara and Tatsumi 1976; Jain and Siede 2013; Masłowska and Pagès 2022). Also, in yeast with extensive replication-associated ssDNA, TS was found to bypass abasic sites upon APOBEC3 or MMS-induced damage (Hoopes et al. 2016; Rosenbaum et al. 2019). Given that FA can induce a variety of similar adducts, the finding that loss of TS does not alter FA-induced mutation rates or sensitivity is surprising. It is possible that bulky FA-induced adducts prevent the strand exchange required for recombination-mediated TS. Further work is needed to determine the impact of TS on FA-induced mutagenesis.

Loss of TLS was shown to significantly reduce the FA-induced mutation rate and further sensitize fork dysregulated strains to FA, suggesting that TLS is the major pathway responsible for the bypass of FA adducts (Fig. 4c and d). These results support our prior findings that Pol ζ is required for the error-prone bypass of FA-induced adducts within areas of ssDNA, and that loss of this pathway can result in an increase in sensitivity, likely due to an abundance of double strand break events (Blouin et al. 2025). Furthermore, Pol ζ-mediated TLS is responsible for most FA-induced mutations even in the absence of fork dysregulation or abundant ssDNA (Fig. 4c). This result might indicate that in replication proficient cells, even small regions of ssDNA are likely targets of FA-induced lesions and mutagenesis.

We observed that the presence of the checkpoint signaling adapters Mrc1 and Rad9 is crucial for cellular survival when exposed to FA; however, defects in this pathway did not alter mutagenesis by FA. We posit that FA likely generates mutagenic adducts on ssDNA, and fork stalling adducts ahead of the replication fork as well (Duxin et al. 2014; Gao et al. 2023). The DNA replication checkpoint is essential to enable repair and bypass of such replication fork blocks. These blocks are not expected to cause increased mutagenesis and instead can culminate in double strand breaks and rearrangements if left unrepaired. Currently, our assay does not directly measure such events. A similar phenomenon was seen in strains deficient for DPC repair, pointing toward DPCs as a likely source of nonmutagenic fork blocking lesions (Blouin et al. 2025). Alternatively, adducts on ssDNA are bypassed by the error-prone TLS machinery resulting in Can^R^ mutagenesis. These data demonstrate that the DNA substrate alters the mutagenic outcome of FA exposure.

## Supplementary Material

iyag189_Supplementary_Data

iyag189_Peer_Review_History

## Data Availability

All data pertaining to this manuscript are presented within the article and supplementary materials. Strains are available upon request. Supplemental material available at GENETICS online.
