# Supplementary material for: Increased replication-associated single-stranded DNA promotes formaldehyde-induced mutagenesis in Saccharomyces cerevisiae
Source: Genetics. 2026 Jul 21;234(3):iyag189. doi: 10.1093/genetics/iyag189 (PMC13732738; doi:10.1093/genetics/iyag189)
Supplement: Supplementary file 1 [file iyag189_peer_review_history.pdf]

GENETICS

Increased replication-associated single-stranded DNA promotes formaldehyde-induced mutagenesis

Thomas Blouin, Charlotte McGuinness, Kierra Marshall, Caroline Bazzle, and Natalie Saini

NOTE: The reviews and decision letters are unedited and appear as submitted by the reviewers.
In extremely rare instances and as determined by a Senior Editor or the EIC, portions of a review may be redacted. If a review is signed, the reviewer has agreed to no longer remain anonymous.
The review history appears in chronological order.

Review Timeline:

Submission Date:	2026-04-29
Editorial Decision:	2026-06-12
Revision Received:	2026-06-30
Accepted:	2026-07-14

1st Editorial Decision: June 12, 2026

June 12, 2026
RE: GENETICS-2026-309431

Dear Dr. Saini:

I am pleased to accept your manuscript titled "Increased replication-associated single-stranded DNA promotes formaldehyde-induced mutagenesis" for publication in GENETICS, pending minor revision.

Please submit your revision along with a brief description of how you modified the manuscript in response to the reviewers' concerns and suggestions (which can be viewed at the bottom of this email. Most important are addressing the concerns from both reviewers with respect to the explanation and interpretation of some of the data. In particular, Reviewer #2 has several comments and suggestions that you should strongly consider and modify the manuscript to address. I expect you should be able to submit a revised manuscript within 30 days. A suitably revised manuscript will be acceptable for publication; I don't expect to send it out for review.

Prior to uploading your revised manuscript please format it according to GENETICS style and ensure you have all required elements. Author Guidelines are at https://academic.oup.com/genetics/pages/author-guidelines#section-12. These guidelines have been updated with new requirements and your careful attention is required to avoid delays at the source file stage post-acceptance.

When revising the ms., please make an effort to shorten it, because that almost always improves a manuscript. We urge authors to heed the advice of Strunk and White: "omit needless words"1. Follow this link to submit the revised manuscript: Link Not Available

Thank you for submitting this nice story to Genetics!

Sincerely,

Jennifer Surtees
Associate Editor
GENETICS

Approved by:
Amy MacQueen
Senior Editor
GENETICS

Reviewer comments:
Reviewer #1 :

The authors aim to determine the underlying mechanism of Formaldehyde (FA)-induced mutagenesis. They provide convincing data that ssDNA serves as a major substrate for FA-induced damage. Furthermore, they determine FA-mediated mutagenesis is dependent on TLS, but not template switching providing mechanistic insight into how cells protect replication forks from FA-induced damage. Overall, the results support the overall conclusions of the manuscript. One results in the manuscript outlined below warrants further clarification.

When examining ssDNA accumulation using CHEF gels (Fig 1D), the chromosome intensity for POL2-deficient samples, and to a lesser extent in POL1-deficient, is significantly reduced compared to WT. The authors postulate the reduced band intensity in POL2-deficient cells is due to excessive fork stalling. However, the authors report there is not a significant growth defect in cells when POL2 or POL1 are down-regulated (Fig 1A). These results are contradictory and need to be resolved.

Reviewer #2 :

Blouin et. al investigate mutagenesis by formaldehyde (FA) and its relationship to ssDNA accumulation in replication mutants

and deficiencies in translesion synthesis. The data here are strong, clear, and support the main claims. The narrative wanders into hyperbole on occasion, which to me was a distraction. I would encourage the authors to be a bit more restrained. It is a nice story, and will certainly be of interest to the DNA damage and mutagenesis fields, after refining the narrative where some aspects need additional attention. The role of template switching, as probed only by ubc13Δ experiments, remains murky, and I think the data there are over-interpreted. The manuscript would benefit from a clearer focus on the relationship between ectopic ssDNA, formaldehyde, TLS, and mutagenesis, rather than peripheral nitty-gritty like REV3 function in the absence of FA, for example.

Specific comments:
The introduction should acknowledge and discuss that FA is well-known to be more reactive on ssDNA where its nucleophilic nitrogen base targets are more exposed. That ssDNA is important for FA mutagenesis is not surprising, but does not diminish the importance of the work presented, so I would be frank about it.

Please put the actual p-values on your plots, rather than asterisks.

Line 101: The increase is 2-fold, and is statistically supported. I would delete the 'significant but modest' qualifier.

Line 117: Is this claim supported? My recollection is that HU treatment, inferred to reveal ssDNA, results in chromosomes that fail to enter CHEF gels, and smears are more typically dsDNA breaks. Given line 132, insert the appropriate reference.

Line 119: While S1 prefers ssDNA, it is not specific for it.

Line 133: In the figure, POL1 - S1 is also clearly lower signal than WT. I suggest making the narrative more consistent with the figure. The details of a semi-quantitative assay in the absence of dox are not particularly important, given that it is pretty clear that polymerase depletion results in more S1-sensitive DNA.

Line 144: I would avoid 'significant'. There is a 5-fold increase, and it is statistically supported.

Line 145: What is a 'weakened fork'? The functions of Mrc1, as you have referenced, are reasonably well-known. You see an increase in FA mutagenesis that is consistent with MRC1 mutants revealing ectopic ssDNA.

Line 153: I think this is an either or, since you have 'not'.

Line 183: There is an increase for wss1Δ, but it doesn't pass your stats test. I agree with your inference that WSS1 is more important for survival than it is for mutagenesis.

Line 207: Why are 'endogenous DNA adducts' invoked here? The data suggest that template switching suppresses mutagenesis in POL2 or POL3 depletion contexts. It could be completely independent of template lesions. The endogenous adduct inference is further undermined when introducing adducts has no effect. I don't find that the ubc13Δ data support the authors' claims.

Line 216: Not a particularly accurate statement. Pol eta? Rev1? The section is perfectly fine as an investigation of the role of pol zeta.

Line 220: I don't understand this statement. For one, the figure clearly shows a decrease in mutagenesis in rev3Δ vs wt. Of course the dynamic range for a decrease is small, but it is well-known that REV3 contributes to mutagenesis. The statement is a distraction from the key result, which is that REV3 generates at least some of the mutations that result when pol depletion/ectopic ssDNA strains are exposed to FA.

The last two paragraphs of the Discussion were, to me, not a strength. The connection of the data presented to a cancer context was not particularly clear. Sure, ssDNA gaps are a thing, and they are mutagenic, but are there indications that they result in increased FA mutagenesis? And the ALDH part was too much conjecture for me.

Methods: I'd like to see the details of the mutation rate calculation. The cited reference doesn't provide a clear path to understanding the methodology used.

We thank the reviewers for their positive review of our study. Below we outline the changes made to the manuscript based on their suggestions. The changes are also marked in red in the markedup copy.

Reviewer #2

The introduction should acknowledge and discuss that FA is well-known to be more reactive on ssDNA where its nucleophilic nitrogen base targets are more exposed. That ssDNA is important for FA mutagenesis is not surprising, but does not diminish the importance of the work presented, so I would be frank about it.

The review is correct that both the lack of template-directed repair and exposing of primary amines typically protected within the interior of the double helix contribute to the higher risk of FA-induced mutagenesis in ssDNA. We have acknowledged this more explicitly in lines 76-78 of the manuscript as follows: “Given that ssDNA lacks template-directed repair and exposes the highly reactive primary amine of guanine and adenine to FA, ssDNA presents as a particularly susceptible genomic substrate for FA-induced mutagenesis (Kawanishi et al., 2014).”

Please put the actual p-values on your plots, rather than asterisks.
We have replaced the asterisks on all graphs with the numerical q-value.

Line 101: The increase is 2-fold, and is statistically supported. I would delete the 'significant but modest' qualifier.
We have deleted the qualifier. Line 183-184 now reads “The wildtype yeast strains demonstrated a 2-fold increase in the Can^R mutation rate upon FA exposure (Figure 1B, Table S2).”

Line 117: Is this claim supported? My recollection is that HU treatment, inferred to reveal ssDNA, results in chromosomes that fail to enter CHEF gels, and smears are more typically dsDNA breaks. Given line 132, insert the appropriate reference.

The reviewer is correct that HU treatment results in chromosomes that fail to enter CHEF gels, mainly due to fork-stalled branch structures and that smearing of bands in a CHEF gel is typically indicative of dsDNA breaks. In our assay, generation of ssDNA via polymerase downregulation followed by S1 digestion results in digestion of the ssDNA regions and smearing of the bands on the CHEF gel which we indicate on line 203. We can be confident that this smearing is due to the digestion of replication-associated ssDNA and not pre-existing dsDNA breaks due to the lack of smearing present in the -S1 condition. To clear up this confusion, we have altered lines 198-200 to read “To confirm ssDNA was being generated in the polymerase downregulation strains, we analyzed genomic DNA via contour clamped homogenous electric field electrophoresis (CHEF), wherein intact chromosomes can be separated as distinct bands.” Separately, on line 203 we indicate that in our assay, smearing would result from S1 digestion of replication-associated ssDNA.

Line 119: While S1 prefers ssDNA, it is not specific for it.

The reviewer is correct that S1 nuclease is not specific to ssDNA as it has been shown to be able to cleave certain rare types of secondary structure in dsDNA. Nonetheless, S1 nuclease activity has been shown to be 10,000x higher on ssDNA than dsDNA, making it a highly selective way to digest ssDNA. We have altered line 201-203 to now read “Each plug was split and half was incubated with S1 nuclease which has been shown to have 10,000x higher nuclease activity on ssDNA than dsDNA for 1.5 hours (Desai and Shankar, 2003).”

Reviewer #2 Line 133: In the figure, POL1 - S1 is also clearly lower signal than WT. I suggest making the narrative more consistent with the figure. The details of a semi-quantitative assay in the absence of dox are not particularly important, given that it is pretty clear that polymerase depletion results in more S1-sensitive DNA.

Reviewer #1 The authors postulate the reduced band intensity in POL2-deficient cells is due to excessive fork stalling. However, the authors report there is not a significant growth defect in cells when POL2 or POL1 are down-regulated (Fig 1A). These results are contradictory and need to be resolved.

Reviewer #1 is correct to point out that our prior wording was contradictory. The spot dilution presented in Figure 1A shows that polymerase downregulation strains do not have altered viability because of the downregulation. Because the spot dilutions are imaged after 48-hours when the strains have had ample time to grow, this assay does not allow us to make any claims about the rate at which these strains grow. We have made this more clear on line 179 by changing the wording to “We did not observe any reduction in viability of yeast strains…”

It is possible that these strains do experience short-term growth defects, potentially due to increased replication fork stalling and ssDNA accumulation, though we do not assay this directly. We have edited the paragraph that starts at line 212 to explain this more clearly.

Line 144: I would avoid 'significant'. There is a 5-fold increase, and it is statistically supported.

We have removed ‘significant’ from line 195.

Line 145: What is a 'weakened fork'? The functions of Mrc1, as you have referenced, are reasonably well-known. You see an increase in FA mutagenesis that is consistent with MRC1 mutants revealing ectopic ssDNA.

We thank the reviewer for noticing ‘weakened fork’ is a poorly defined term. We meant the reader to interpret a weak fork as one that has somehow lost normal regulation, resulting in the accumulation of ssDNA. To make this more clear, we have defined dysregulated replication forks in the introduction on line 89-92 stating: “Loss of this pathway or alterations to the normal replisome composition, like replicative polymerase downregulation or loss of critical replisome proteins (Mrc1/CLASPIN, Tof1/Timeless, Csm3/Tipin) have been shown to cause fork dysregulation resulting in abundant replication-associated ssDNA that is prone to breakage and/or mutagenesis”. Following this, we have replaced ‘weakened fork’ on lines 189 and 229 with ‘dysregulated forks’.

Line 153: I think this is an either or, since you have 'not'.

We have made the suggested edit.

Line 183: There is an increase for wss1∆, but it doesn't pass your stats test. I agree with your inference that WSS1 is more important for survival than it is for mutagenesis.

We added ‘no significant increase’ to line 267 because the reviewer is correct that there was a minor increase.

Line 207: Why are 'endogenous DNA adducts' invoked here? The data suggest that template switching suppresses mutagenesis in POL2 or POL3 depletion contexts. It could be completely independent of template lesions. The endogenous adduct inference is further undermined when introducing adducts has no effect. I don't find that the ubc13∆ data support the authors' claims.

While the mutation rates of *TetR-POL2-ubc13∆* and *TetR-POL3-ubc13∆* are significantly higher than *TetR-POL2* and *TetR-POL3* we disagree with the reviewer that the only role for template switching is in these polymerase depletion contexts. There is a 7-fold increase in the CanR mutation rate in *ubc13∆* strains that have no dysregulated replication. This increase in strains with unperturbed replication suggests that template switching is important in bypassing some types of endogenously occurring damage. It is possible that the type of adduct template switching bypasses in the absence of exogenous damage is different than FA-induced adducts which seem to be incompatible with template switching. However, since we have no way to assay endogenously produced damage, we have removed ‘endogenous DNA adducts’ from the paragraph beginning on line 280.

Line 216: Not a particularly accurate statement. Pol eta? Rev1? The section is perfectly fine as an investigation of the role of pol zeta.

The reviewer is correct that there are other TLS polymerases that may be available for bypassing FA-induced adducts. As such, we have removed the line “Translesion synthesis (TLS) function in yeast is dependent on Pol ζ (Haracska et al., 2003; Johnson et al., 2000; Martin and Wood, 2019; Prakash et al., 2005; Sale, 2013; Shachar et al., 2009). However, previous work from our lab has shown that Pol ζ is required for the mutagenic bypass of FA-induced adducts and that in the absence of Pol ζ persistent stalling (TLS inactivity) results in chromosome breakage events, suggesting that Pol ζ is required for the TLS-mediated bypass of FA-induced adducts. We have added a line describing these results starting on line 307 “These results agree with prior work from our lab showing that Pol ζ is required for the mutagenic bypass of FA-induced adducts within ssDNA and that in the absence of Pol ζ persistent stalling due to inefficient TLS results in chromosome breakage events (Blouin et al., 2025)”.

Line 220: I don't understand this statement. For one, the figure clearly shows a decrease in mutagenesis in rev3Δ vs wt. Of course the dynamic range for a decrease is small, but it is well-known that REV3 contributes to mutagenesis. The statement is a distraction from the key result, which is that REV3 generates at least some of the mutations that result when pol depletion/ectopic ssDNA strains are exposed to FA.

The reviewer is correct that the *rev3*Δ strains grown on YPD have a slightly lower mutation rate than what is seen in the wildtype grown on YPD. This decrease however is less than 2-fold and nonsignificant. We observe a significant increase in the mutation rate of strains with nonfunctional template switching when grown on YPD. These two results together led us to conclude that the error-free template switching pathway is the cells preferred way to deal with endogenously produced DNA damage, and that translesion synthesis is reserved for exogenously produced DNA damage.

The last two paragraphs of the Discussion were, to me, not a strength. The connection of the data presented to a cancer context was not particularly clear. Sure, ssDNA gaps are a thing, and they are mutagenic, but are there indications that they result in increased FA mutagenesis? And the ALDH part was too much conjecture for me.

We thank the reviewer for the comment and agree that the final two paragraphs of the conclusion were a bit too speculative. We have removed these from the revised version to keep the discussion more concise.

Methods: I'd like to see the details of the mutation rate calculation. The cited reference doesn't provide a clear path to understanding the methodology used.

We have added more detail regarding the mutation rate calculation to line 137 of the manuscript.

July 14, 2026
RE: GENETICS-2026-309431R1

Dr. Natalie Saini
Medical University of South Carolina
Biochemistry and Molecular Biology
173 Ashley Ave
BS518C
Charleston, South Carolina 29425-8908

Dear Dr. Saini:

Congratulations, your manuscript titled "Increased replication-associated single-stranded DNA promotes formaldehyde-induced mutagenesis" is accepted for publication in GENETICS! I appreciate your thoughtful responses to the reviewer comments. Many thanks for submitting your research to the journal.

To Proceed to Publication:
1. Format your article according to GENETICS style: https://academic.oup.com/genetics/pages/author-guidelines

2. Ensure that you comply with data and community resource citation guidelines:
https://academic.oup.com/genetics/pages/author-guidelines#section-5-9-2

3. Upload your final files at https://genetics.msubmit.net

4. Add oupsupport@scipris.com and genetics.oup@novatechset.com (or the domains @scipris.com and @novatechset.com) to your email program's "safe senders" list. You will be contacted by both at various points during the production process.

Notes:
- Your currently-accepted manuscript (unedited, as submitted, reviewed, and accepted) will be published at GENETICS and deposited into PubMed as an Advance Access article. Notify sourcefiles@thegsajournals.org before signing your license if you do not wish to publish your article via Advance Access.

- We invite you to submit an original color figure related to your paper for consideration as cover art. Please note that submissions created with AI image generation tools are not eligible. Please email your submission to the editorial office or upload it with your final files. You can submit a small-sized image for evaluation, and if selected, the final image must be a TIFF file 2513px wide by 3263px high (8.375 by 10.875 inches; resolution of 600ppi). Please avoid graphs and small type.

- After files are sent to Oxford University Press we use SciPris to manage article licensing and payment. If you do not have a SciPris account, you will receive an email from no-reply@scipris.com to sign up to use Oxford University Press' author portal. After logging in, follow the online instructions to sign your license and arrange any payment due.

If you have any questions or encounter any problems while uploading your accepted manuscript files, please email the editorial office at sourcefiles@thegsajournals.org.

Sincerely,

Jennifer Surtees
Associate Editor
GENETICS

Approved by:
Amy MacQueen
Senior Editor
GENETICS
