# Supplementary material for: Increased replication-associated single-stranded DNA promotes formaldehyde-induced mutagenesis in Saccharomyces cerevisiae
Source: Genetics. 2026 Jul 21;234(3):iyag189. doi: 10.1093/genetics/iyag189 (PMC13732738; doi:10.1093/genetics/iyag189)
Supplement: Supplementary file 2 [file iyag189_supplementary_data.zip › Supplemental_Figures_GENETICS-2026-309431.pdf]

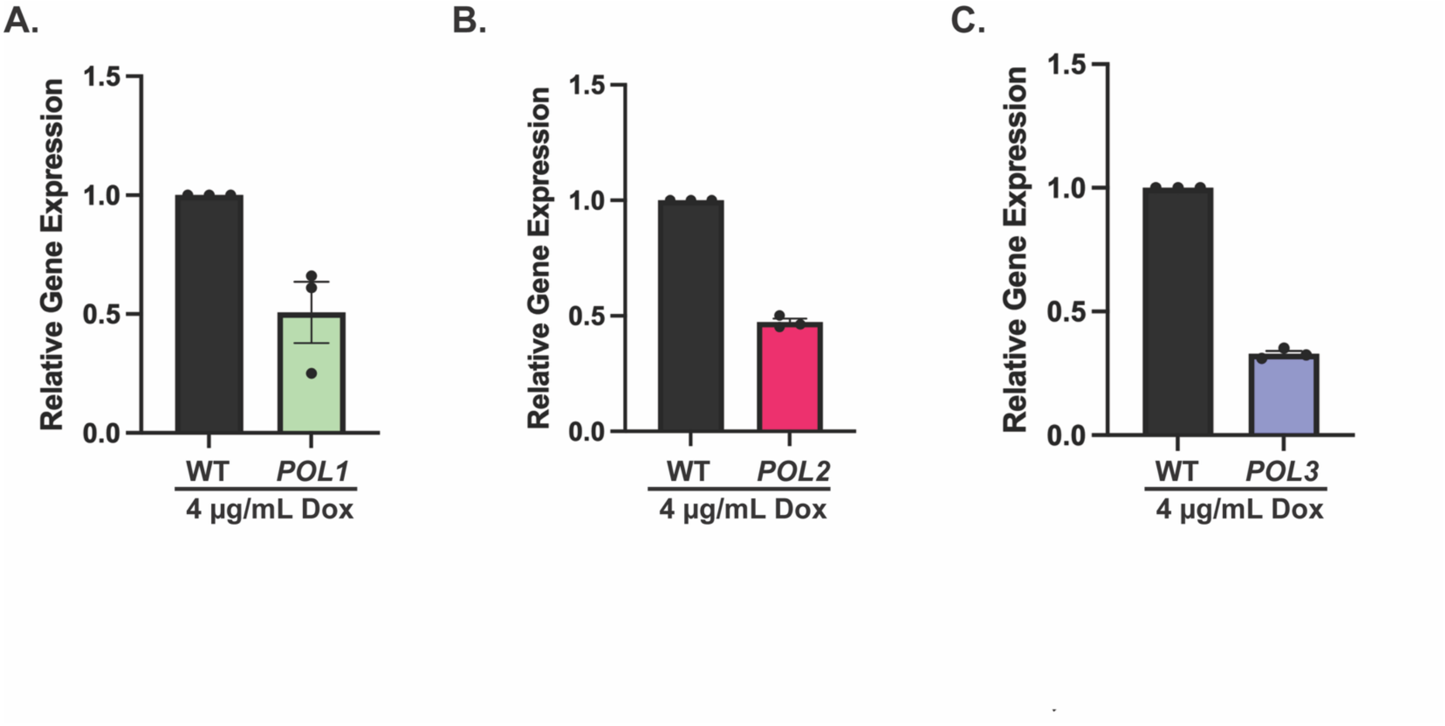

<u>Figure S1: RT-qPCR of *TetR-POL* strains.</u> Gene expression was monitored by RT-qPCR for the replicative polymerases under control of tetracycline repressible *tetO*$_7$ promoters. Expression was compared against the *POL* gene under the control of its endogenous (WT) promoter. Mean expression levels and standard error of the mean are depicted. Data for panels B and C are originally from Porcher et al., 2025.

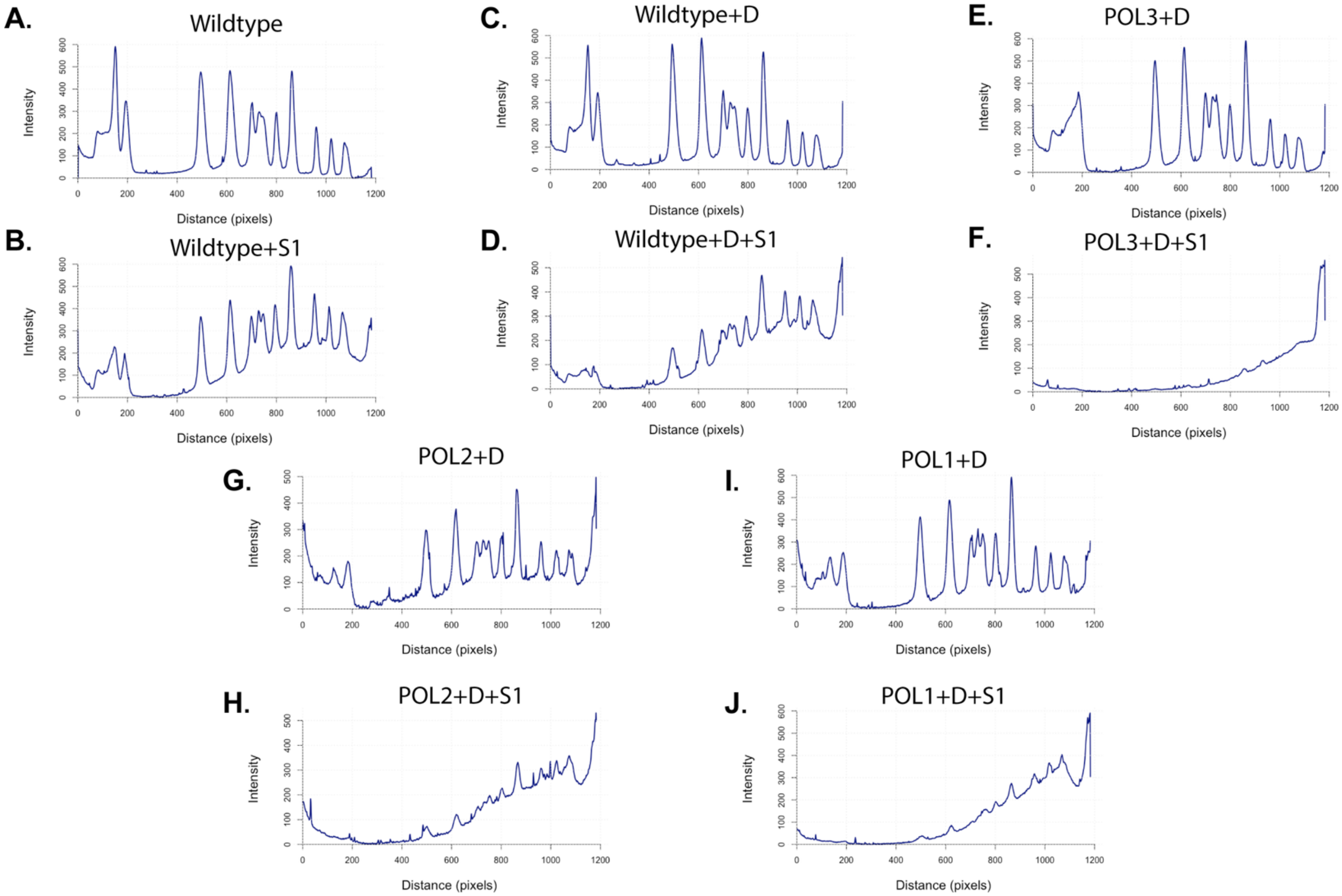

Figure S2: Peak intensity profile of polymerase downregulation CHEF gel. Peak intensity profiles of each lane in the CHEF gel featured in Figure 1D. Intensity plots were generated in ImageJ.

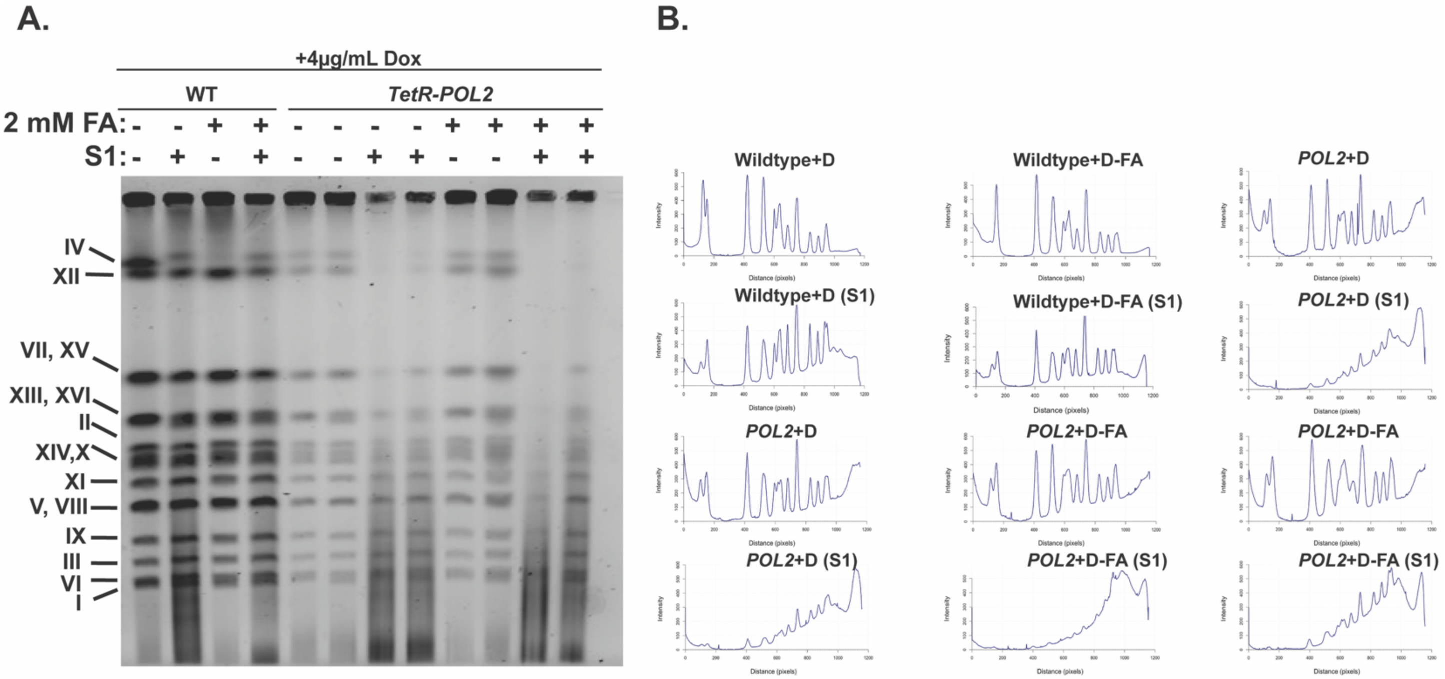

Figure S3: Formaldehyde treatment itself does not result in higher levels of replication associated ssDNA. A. Representative CHEF gel of wildtype or *TetR-POL2* strains grown in the presence of doxycycline and/or FA exposed to the ssDNA specific S1 nuclease. B. Peak intensity profiles of each lane in the CHEF gel.

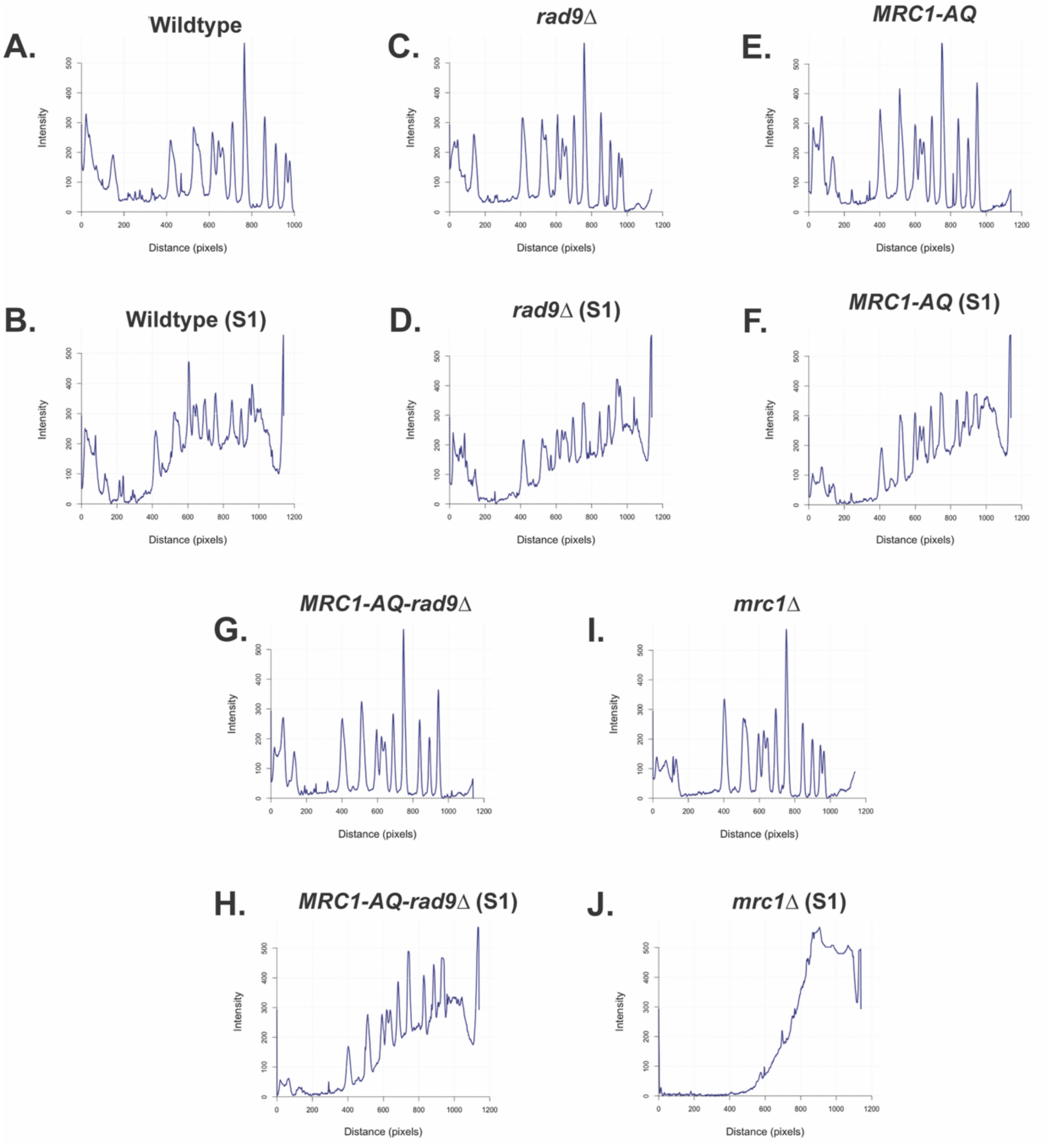

Figure S4: Peak intensity profile of Mrc1 and checkpoint signaling mutant CHEF gel. Peak intensity profiles of each lane in the CHEF gel featured in Figure 2C. Intensity plots were generated in ImageJ.
